# Duality in Binocular Rivalry: Distinct Sensitivity of Percept Sequence and Percept Duration to Imbalance between Monocular Stimuli

**DOI:** 10.1371/journal.pone.0006912

**Published:** 2009-09-07

**Authors:** Chen Song, Haishan Yao

**Affiliations:** 1 Institute of Neuroscience, State Key Laboratory of Neuroscience, Shanghai Institutes for Biological Sciences, Chinese Academy of Sciences, Shanghai, China; 2 Department of Biomedical Engineering, Shanghai Jiao Tong University, Shanghai, China; Macquarie University, Australia

## Abstract

**Background:**

Visual perception is usually stable and accurate. However, when the two eyes are simultaneously presented with conflicting stimuli, perception falls into a sequence of spontaneous alternations, switching between one stimulus and the other every few seconds. Known as binocular rivalry, this visual illusion decouples subjective experience from physical stimulation and provides a unique opportunity to study the neural correlates of consciousness. The temporal properties of this alternating perception have been intensively investigated for decades, yet the relationship between two fundamental properties - the sequence of percepts and the duration of each percept - remains largely unexplored.

**Methodology/Principal Findings:**

Here we examine the relationship between the percept sequence and the percept duration by quantifying their sensitivity to the strength imbalance between two monocular stimuli. We found that the percept sequence is far more susceptible to the stimulus imbalance than does the percept duration. The percept sequence always begins with the stronger stimulus, even when the stimulus imbalance is too weak to cause a significant bias in the percept duration. Therefore, introducing a small stimulus imbalance affects the percept sequence, whereas increasing the imbalance affects the percept duration, but not vice versa. To investigate why the percept sequence is so vulnerable to the stimulus imbalance, we further measured the interval between the stimulus onset and the first percept, during which subjects experienced the fusion of two monocular stimuli. We found that this interval is dramatically shortened with increased stimulus imbalance.

**Conclusions/Significance:**

Our study shows that in binocular rivalry, the strength imblanace between monocular stimuli has a much greater impact on the percept sequence than on the percept duration, and increasing this imbalance can accelerate the process responsible for the percept sequence.

## Introduction

**Figure 1:**
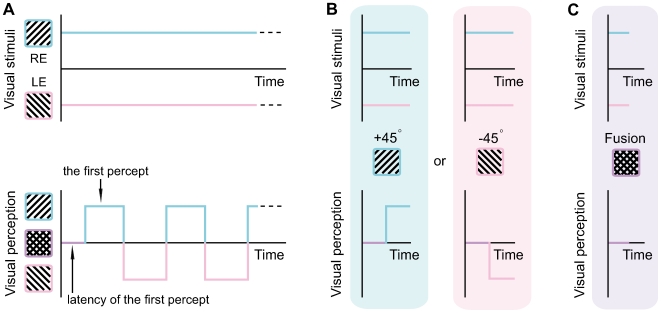
Schematic depiction of binocular rivalry and experimental paradigms. (A) To induce binocular rivalry, a pair of orthogonal gratings (tilted +45° and −45° away from the vertical) were separately presented to the two eyes of the human subjects, and subjects experienced the alternating dominance between one grating and the other. Between the stimulus onset and the first percept, there existed a short period during which subjects experienced the fusion of the two gratings, rather than the complete dominance of one grating over the other. (B) and (C) To measure the first percept and its latency, rivalry stimuli were briefly presented for a duration varied from 10 msec to 500 msec, and subjects reported their perception through a three-alternative forced choice (+45° grating, the −45° grating, or the fusion of two gratings). The trails in which subjects reported the fusion perception were taken into account only when we calcualted the latency of the first percept, and different conditions of stimulus duration were combined in the analyses except for calculating this latency.

Overlapping visual fields of the two eyes allow the brain to reconstruct the three-dimensional structure of the visual world. In daily vision, the left and right eyes receive slightly different views of the same object, and the resulting binocular disparity gives rise to the perception of depth [1]. When the two eyes are simultaneously presented with conflicting stimuli, however, something fascinating can happen: rather than forming a stable vision, the two stimuli can compete for visual dominance, with perception alternating between one stimulus and the other every few seconds (Figure 1A) [2], [3]. Because the subjective perception keeps fluctuating while the visual stimuli remain invariant, this binocular rivalry phenomenon is widely used as a tool for exploring the neural correlates of consciousness [4]–[8].

Given its importance, the neural mechanism underlying this fascinating phenomenon has long been a central theme in vision research. Originally, disputes exist between the ‘eye rivalry’ theory that binocular rivalry arises from low-level competition between monocular neurons [9], and the ‘stimulus rivalry’ theory that binocular rivalry reflects high-level competition between stimulus representations [10]. Recently, however, converging lines of evidence point to a hybrid theory that binocular rivalry entails multiple processes operating at different levels of the visual hierarchy [11]–[13].

In addition to these theoretical advancements, progress has been made on studying the hallmark of binocular rivalry - the temporal dynamics of rivalry perception. Early works on rivalry dynamics (for a review, see [14]) usually focus on the alternating perception during sustained presentation of rivalry stimuli (Figure 1A). They have shown that the alternations in perception and the duration of each percept can be affected by stimulus strength (e.g., contrast, luminance, spatial frequency). Specifically, the strength imbalance between two monocular stimuli can lead to bias in the percept duration, such that the stronger stimulus enjoys a longer percept duration [15]. Recently, with more and more studies using the paradigm of brief presentation (Figure 1B) [16]–[27], the distinctions between the perception at rivalry onset and the perception during prolonged rivalry have come to light. The perception at rivalry onset tends to be dominated by the stimulus in the preferred eye [16], [17], the stimulus in a specific color (probably the preferred color) [18], the stimulus with high contrast [19]–[21], the stimulus with cued attention [20], the stimulus stored in sensory memory [21]–[24], and the stimulus with no pre-adaptation [18], [25]–[27]. In contrast, the perception during prolonged rivalry is distributed between two monocular stimuli in a much more balanced fashion [17], [18], [20], [22]. These findings demonstrate that the percept sequence in binocular rivalry is biased to begin with the stimulus of greater effective strength, and the degree of bias is larger than that in the percept duration.

Based on these empirical observations, Carter and Cavanagh have proposed that the percept sequence and the percept duration are fundamentally different (i.e., the perception at rivalry onset is fundamentally different from the perception during prolonged rivalry [18]). To quantitatively examine the relationship between the percept sequence and the percept duration, we varied the contrast imbalance between two monocular stimuli and compared its effect on the percept sequence versus the percept duration. We found that varying the contrast imbalance from 0% to 20% affects the percept sequence, whereas varying the contrast imbalance from 20% to 80% affects the percept duration, but not vice versa. This demonstrates that, compared with the percept duration, the percept sequence is far more sensitive to the contrast imbalance between monocular stimuli. To understand the mechanism of such high sensitivity in the percept sequence, we further measured the latency of the first percept (i.e., the interval between the stimulus onset and the first percept, Figure 1A). We found that the increase in contrast imbalance dramatically shortens this latency, indicating that the process responsible for the percept sequence is accelerated with increased stimulus imbalance. Therefore, this process is likely to involve the comparison between two monocular stimuli to establish the degree of binocular correspondence, and the inhibition of the weaker stimulus upon registration of binocular incompatibility.

## Results

Binocular rivalry was induced by a pair of orthogonal gratings (tilted +45° and −45° away from the vertical) presented separately to the two eyes of the human subjects. The contrast of the two gratings was randomly chosen from (50% vs. 50%), (60% vs. 40%), (70% vs. 30%), (80% vs. 20%), and (90% vs. 10%) for each presentation. Because the sequence of percepts is determined by the first percept (Figure 1A), we utilized the paradigm of brief presentation [28] to measure the first percept and its latency. Rivalry stimuli were briefly presented for a duration varied from 10 msec to 500 msec, and subjects reported which of the two stimuli (the +45° grating or the −45° grating) was dominant (Figure 1B). Subjects were given an extra choice if they experienced the fusion of the two gratings (Figure 1C), but these trials were taken into account only when we calculated the latency of the first percept. Different conditions of stimulus duration were combined in the analyses except for calculating the latency of the first percept. To measure the duration of each percept, we used the paradigm of sustained presentation (Figure 1A). Subjects tracked their perception (the +45° grating or the −45° grating) during a 100-sec sustained presentation of rivalry stimuli.

**Figure 2:**
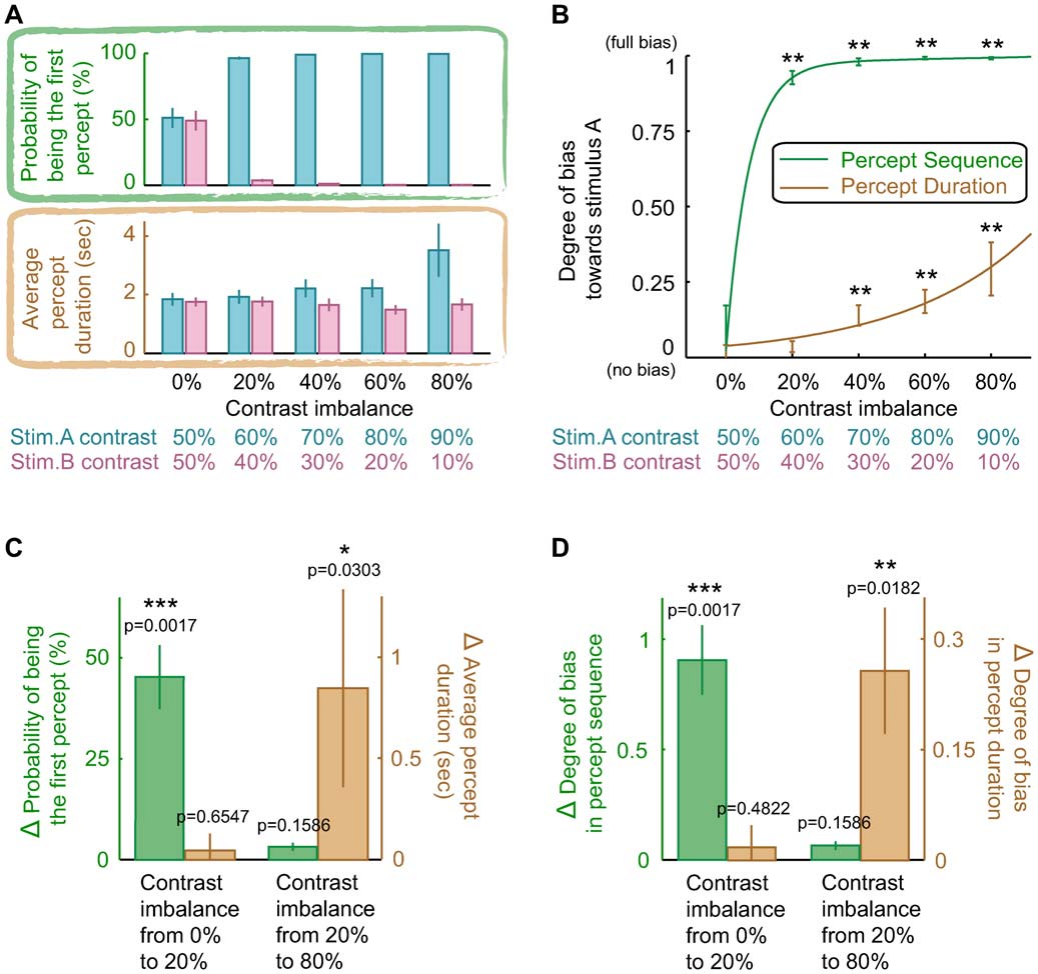
Influence of contrast imbalance on percept sequence versus percept duration. (A) and (B) There is no bias in the percept sequence (Wilcoxon signed-rank test, p>0.9) when the contrast imbalance is 0%. When the contrast imbalance is at or above 20%, the percept sequence is biased to begin with the higher-contrast stimulus (**, Wilcoxon signed-rank test, p<0.02). There is no bias in the percept duration (Wilcoxon signed-rank test, p>0.1) when the contrast imbalance is at or below 20%. When the contrast imbalance is at or above 40%, the percept duration is biased and the higher-contrast stimulus enjoys a longer percept duration (**, Wilcoxon signed-rank test, p<0.02). Compared with the percept duration, the percept sequence can detect a smaller contrast imbalance and has a much larger degree of bias. Error bars represent 1 SEM (n = 7). (C) When the contrast imbalance increases from 0% to 20%, the probability of being the first percept significantly changes (***, Kruskal-Wallis test, p<0.002), but the average percept duration does not (Kruskal-Wallis test, p>0.5). When the contrast imbalance increases from 20% to 80%, the average percept duration significantly changes (*, Kruskal-Wallis test, p<0.05), but the probability of being the first percept does not (Kruskal-Wallis test, p>0.1). Error bars represent 1 SEM (n = 7). (D) When the contrast imbalance increases from 0% to 20%, the degree of bias in the percept sequence significantly increases (***, Kruskal-Wallis test, p<0.002), but that in the percept duration does not (Kruskal-Wallis test, p>0.1). When the contrast imbalance increases from 20% to 80%, the degree of bias in the percept duration significantly increases (**, Kruskal-Wallis test, p<0.02), but that in the percept sequence does not (Kruskal-Wallis test, p>0.1). Error bars represent 1 SEM (n = 7).

Figure 2A shows the effect of contrast imbalance on the percept sequence (Figure 2A, green box) versus the percept duration (Figure 2A, brown box). Because the percept sequence is determined by the first percept (Figure 1A), we calculated the probability of being the first percept for each stimulus. As expected, the two stimuli with balanced contrast have equal probability to be the first percept, and have equal percept duration as well. When they are imbalanced, the percept sequence is significantly biased such that it begins with the stronger (e.g., higher-contrast) stimulus with a probability greater than 95%, and the percept duration is also biased towards the stronger stimulus. To evaluate whether the percept sequence or the percept duration is more sensitive to the imbalance between monocular stimuli, we directly compared their degrees of bias for each contrast imbalance. As can be seen from Figure 2B, while the contrast imbalance of 20% is strong enough to cause a significant bias in the percept sequence (Wilcoxon signed-rank test, p<0.02), it is too weak to cause bias in the percept duration (Wilcoxon signed-rank test, p>0.1). Moreover, the degree of bias in the percept sequence is higher than 0.9 for the smallest contrast imbalance tested, whereas that in the percept duration is lower than 0.3 even for the largest contrast imbalance tested. Capable of detecting a small imbalance with a large bias, the percept sequence undoubtedly is much more sensitive to the stimulus imbalance than does the percept duration.

To investigate the relationship between the percept sequence and the percept duration, we further compared the shape of their sensitivity curves (Figure 2B). We found that the degree of bias in the percept duration increases gradually with the stimulus imbalance. By contrast, the degree of bias in the percept sequence is saturated at the smallest imbalance tested. Consequently, varying the contrast imbalance from 0% to 20% affects the degree of bias in the percept sequence but not that in the percept duration, and varying the contrast imbalance from 20% to 80% affects the degree of bias in the percept duration but not that in the percept sequence (Figure 2D). In a similar vein, we found that varying the contrast imbalance from 0% to 20% and from 20% to 80% have distinct impacts on the average percept duration and the probability of being the first percept (Figure 2C). Therefore, change in the percept sequence does not necessarily correspond to change in the percept duration.

**Figure 3:**
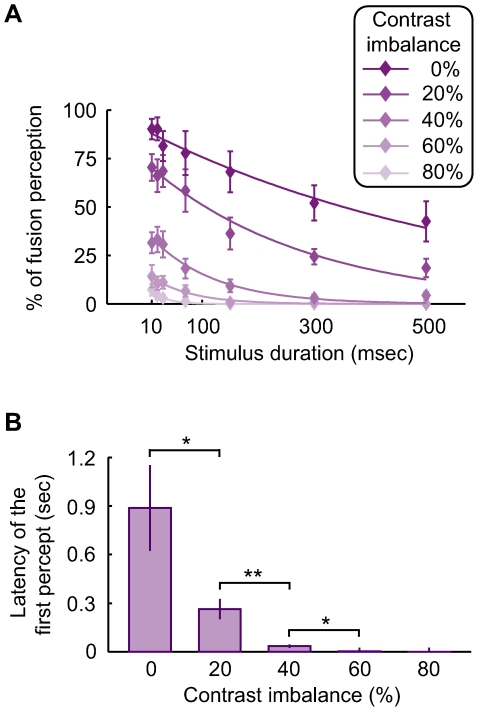
Influence of contrast imbalance on the latency of the first percept. (A) For each contrast imbalance, the latency of the first percept was quantified as the stimulus duration that corresponded to 33% of fusion perception. Error bars represent 1 SEM (n = 7). (B) The latency of the first percept shortens as the contrast imbalance increases from 0% to 20% (*, Wilcoxon rank-sum test, p<0.05), from 20% to 40% (**, Wilcoxon rank-sum test, p<0.02), and from 40% to 60% (*, Wilcoxon rank-sum test, p<0.05). Error bars represent 1 SEM (n = 7).

What mechanisms are responsible for the sensitivity difference between the percept sequence and the percept duration? As a topic that has been extensively studied for decades, the alternations in rivalry perception and the duration of each percept are generally considered to be mediated by neural adaptation [25], [29], [30]. In contrast, little is known about the process responsible for the sequence of rivalry perception. Nonetheless, Blake [14] has proposed that, the brain selects the stimulus for perception at rivalry onset through the comparison between two monocular stimuli to establish the degree of binocular correspondence. To examine this hypothesis, we measured the latency of the first percept (Figure 1A). We reasoned that the increase in stimulus imbalance should facilitate the comparison process and thereby shorten this latency. Because the subjects experienced the fusion of two monocular stimuli during the latency period [28] (Figure 1A), we calculated the percentage of fusion perception for different stimulus durations (Figure 3A), and estimated this latency as the stimulus duration that corresponded to 33% of fusion perception (see Data analyses in Methods). Interestingly, increasing the contrast imbalance does shorten the latency of the first percept (Figure 3B; Wilcoxon rank-sum test, p<0.05). Thus, the process responsible for the percept sequence is accelerated with increased stimulus imbalance, supporting the hypothesis that this process involves the comparison between two monocular stimuli.

## Discussion

Our study shows that in binocular rivalry, the percept sequence is far more sensitive to the strength imbalance between monocular stimuli than does the percept duration, and increasing the stimulus imbalance accelerates the process responsible for the percept sequence. These results suggest that binocular rivalry may involve dual stages of process: an initial stage for the percept sequence and a sustained stage for the percept duration. During the initial stage, subjects experience the fusion of two monocular stimuli [28], while the visual system establishes the degree of binocular correspondence and inhibits the weaker stimulus upon registration of binocular incompatibility. During the sustained stage, subjects experience the alternation between two monocular stimuli [2], [3], while the visual system adapts to the dominant stimulus and switches dominance to the suppressed stimulus upon the break of balance [25], [29], [30].

Actually, some theoretical studies have already noticed that the selection of the perceptually dominant stimulus at rivalry onset (which determines the percept sequence) and the alternations in perceptual dominance during prolonged rivalry (which determine the percept duration) should be distinguished [14], [31], [32]. Among them, Blake [14] and Noest et al. [31] proposed that the selection and the alternation should be counted as two separate neural processes. Hohwy et al. [32] proposed that the selection and the alternation could both be explained by Beyesian inference regardless of their detailed neural mechanisms. Specifically, Hohwy et al. [32] proposed that the stimulus with higher prior probability (i.e., the stimulus which is more likely to appear according to the viewing history; e.g, as the face stimulus is encountered most frequently in daily life, it may have higher prior probability than other stimuli) is selected for perception at rivalry onset, and perception alternates because the brain tries to minimize the prediction error between the high-level prediction (i.e., the current percept, the currently dominant stimulus) and the low-level unexplained signal (i.e., the currently suppressed stimulus). Noest et al. [31] modeled the selection process as how trajectories diverge when approaching the saddle point between two coexisting attractors that encode two potential percepts, and modeled the alternations in perception as resulting from adaptation-driven destabilization of the currently active attractor. In addition to these abstract frameworks, Blake [14] has proposed some concrete mechanisms, that the selection process involves the comparison of information presented to the two eyes to establish the degree of binocular correspondence, and the alternation process involves neural adaptation that weakens the neural responses to the dominant stimulus.

Despite the apparent differences among these theories, they all lead to the prediction that the perception at rivalry onset and the percept during prolonged rivalry shall have different characteristics. Particularly, the perception at rivalry onset shall be well suited to probe the incompatibility (e.g., strength imbalance) between two monocular stimuli. Indeed, several studies before ours have shown that the perception at rivalry onset and the perception during prolonged rivalry have different degrees of bias: the perception at rivalry onset is trapped to the stimulus of high signal strength [16]–[27], whereas the perception during prolonged rivalry is distributed between two monocular stimuli [17], [18], [20], [22]. They have shown that, the high signal strength could be attributed to the preference to eye-of-presentation [16], [17], the preference to color-of-presentation [18], the high contrast [19]–[21], the allocation of attention [20], the storage in sensory memory [21]-[24], whereas the low signal strength could be attributed to the effect of neural adaptation [18], [25]–[27]. However, none of these studies have quantitatively examined the relationship between the perception at rivalry onset (the percept sequence) and the perception during prolonged rivalry (the percept duration). Here, by quantifying their sensitivity to the strength imbalance between two monocular stimuli, we show that the percept sequence is superior in detecting the strength imbalance, because it not only detects the imbalance with a larger bias [17], [18], [20], [22], but also detects smaller imbalance, than the percept duration does. Clearly, our study provides empirical evidence for these theories [14], [31], [32] that binocular rivalry involves an initial selection process for the percept sequence and a sustained alternation process for the percept duration.

Furthermore, among these theories [14], [31], [32], Blake's [14] has proposed a concrete mechanism for how the brain selects the perceptually dominant stimulus at rivalry onset. According to this theory, the selection process involves the comparison of information presented to the two eyes to establish the degree of binocular correspondence. Notably, early studies on the relationship between binocular fusion and binocular rivalry have suggested something similar, that the visual system needs to examine the compatibility of two monocular stimuli at stimulus onset [33], [34], and that compatible stimuli leads to binocular fusion or binocular disparity and incompatible stimuli to binocular rivalry [35], [36]. Our finding that the latency of the first percept shortens with increased stimulus imbalance (i.e., stimulus incompatibility) provides additional support for this hypothesis. Therefore, it is likely that the initial selection process for the percept sequence is shared, at least in part, among different types of binocular vision, and the sustained alternation process for the percept duration is unique to bistable vision.

Our study provides firmed evidence that, in binocular rivalry, the sequence of percepts and the duration of each percept show distinct sensitivity to the strength imbalance between monocular stimuli. Moreover, the process responsible for the percept sequence accelerates with increased incompatibility between two monocular stimuli, and may involve the comparison of two monocular stimuli to establish the degree of binocular correspondence. Further studies are needed to reveal whether the sensitivity difference between the percept sequence and the percept duration is universal in all types of bistable vision [3], and how the process responsible for the percept sequence differs among different types of bistable vision.

## Materials and Methods

### Subjects

Seven right-handed subjects with normal or corrected-to-normal vision participated in this study. Apart from one of the authors (CS), all subjects were naive to the aims of the experiments and received payment for participation. This study was undertaken with the understanding and written consent of each subject, and the approval from the Academic Board of Institute of Neuroscience, Chinese Academy of Sciences.

### Apparatus and stimuli

A pair of gray-scale sinusoidal gratings (2° in diameter, spatial frequency 3.5 cycles/°), oriented at +45° and −45° away from the vertical, were presented on the two halves of a calibrated CRT Monitor (Viewsonic P225, 22″, 1024×768 resolution, 100 Hz refresh rate). To aid binocular convergence, each grating was centered in a circle (inner and outer diameters of 2° and 2.24°), and a rectangle (3.4°×5°) with Nonius lines (0.4°×0.14°) on its edge. The experiments, programmed with MATLAB Psychtoolbox [37], were conducted in a darkened room with the monitor providing the only significant source of light. The average luminance of the gratings was equal to that of the uniform gray background (26.1 cd/m^2^), and the convergence clues were relatively dark (1.6 cd/m^2^) compared to the gratings. Subjects viewed visual stimuli through a mirror stereoscope with a chin rest, and reported their perception by pressing the assigned keys on a keyboard.

### Experimental procedures

#### Brief-presentation experiment

The contrast of the +45° and −45° gratings was (10% vs. 90%), (20% vs. 80%), (30% vs. 70%), (40% vs. 60%), (50% vs. 50%), (60% vs. 40%), (70% vs. 30%), (80% vs. 20%), or (90% vs. 10%). The stimulus duration was 10 msec, 20 msec, 30 msec, 70 msec, 150 msec, 300 msec, or 500 msec. To minimize the effect of sensory memory [24], [38], the stimulus contrast, duration, and eye-of-presentation were randomly chosen for each presentation (trial). Each combination of contrast and duration was tested for 16 trials, and the experiment involved a total of 1008 trials. Subjects reported their perception through a three-alternative forced choice (the +45° grating, the −45° grating, or the fusion of two gratings) after each presentation (trial). A preliminary experiment was conducted to ensure that the stimuli (the +45° grating, the −45° grating, and the fusion of two gratings) under each combination of contrast and duration were far beyond the detection threshold (correct answers were given for more than 90% of the trials) and subjects understood the instructions correctly.

#### Sustained-presentation experiment

The contrast of the +45° and −45° gratings was (10% vs. 90%), (20% vs. 80%), (30% vs. 70%), (40% vs. 60%), (50% vs. 50%), (60% vs. 40%), (70% vs. 30%), (80% vs. 20%), or (90% vs. 10%). The stimulus duration was 100 sec. The stimulus contrast and eye-of-presentation were randomly chosen for each presentation (trial). Each contrast was tested for 2 trials, and the experiment involved a total of 18 trials. Subjects reported their perception (the +45° grating or the −45° grating) when their perception altered. Subjects were not instructed to report the fusion of two gratings, because that perception lasted for a rather short duration and may cause inaccurate report.

### Data analyses

#### Quantify the sensitivity to the stimulus imbalance

To evaluate whether the percept sequence or the percept duration is more sensitive to the imbalance between monocular stimuli, we made a direct comparison between their degrees of bias. For the percept sequence, the degree of bias towards stimulus A was calculated as (P_A_ − P_B_) / (P_A_ + P_B_), where P_A_ and P_B_ denote the probability of being the first percept for stimulus A and stimulus B, respectively. For the percept duration, the degree of bias towards stimulus A was calculated as (D_A_ − D_B_) / (D_A_ + D_B_), where D_A_ and D_B_ denote the average percept duration for stimulus A and stimulus B, respectively. Therefore, the degree of bias is 0 if there is no bias at all, and the degree of bias is 1 in the case of full bias. The degree of bias was calculated for each contrast imbalance, and the data were fitted with exponential functions (Figure 2B).

To investigate the relationship between the percept sequence and the percept duration, we further examined the effect of increasing the contrast imbalance from 0% to 20% and from 20% to 80% on them. Suppose that {Y_1_, Y_2_, Y_3_, Y_4_, Y_5_} denote the degree of bias in the percept sequence at the contrast imbalance {0%, 20%, 40%, 60%, 80%}, Δ degree of bias in the percept sequence was calculated as | Y_2_ − Y_1_ | and as | Y_5_ − Y_2_ |; Kruskal-Wallis test was conducted between {Y_1_, Y_2_} and among {Y_2_, Y_3_, Y_4_, Y_5_}. We performed similar analyses for the degree of bias in the percept duration, the percept sequence (i.e., the probability of being the first percept), and the percept duration (i.e., the average percept duration). Because the average percept duration is different for stimulus A and stimulus B, we performed separate analyses for each of them. We took the average of their Δ average percept duration, and the minimum of their Kruskal-Wallis test p-value as the final result.

#### Measure the latency of the first percept

To measure the latency of the first percept, we calculated the percentage of fusion perception for each stimulus duration, and fitted the data with exponential functions (Figure 3A). Because subjects reported their perception through a three-alternative choice (the +45° grating, the −45° grating, or the fusion of two gratings), the latency was quantified as the stimulus duration that corresponded to 33% of fusion perception.

#### Eliminate the impact of eye preference

As a result of eye preference, rivalry perception is biased towards the stimulus presented to the preferred eye [16], [17], e.g., the percept sequence tends to begin with this stimulus, and this stimulus tends to have a longer percept duration. To statistically eliminate the impact of eye preference, the contrast value at which the two eyes reached equal predominance was set to replace the contrast value of (50% vs. 50%), i.e., the difference between these two contrast values was subtracted from the original contrast value. We performed this contrast transformation for each individual subject. The data from all subjects were combined and binned at an interval of 10%, i.e., the contrast values within the range of (10 × n − 5)% to (10 × n + 5)% were replaced with (10 × n)% (n  =  1, 2, 3, …, 9).

#### Examine the impact of sensory memory

To examine whether the results of the brief-presentation experiment are affected by the sensory memory [24], [38], we divided the original data into three blocks. Each block contained the trials in which the previous perception was the right-eye stimulus, the left-eye stimulus, and the fusion of two stimuli, respectively. Results in these three blocks did not show significant difference (Kruskal-Wallis test, p>0.5). Because the sensory memory could be attributed to eye-of-origin or stimulus-of-origin [23], we also divided the original data according to whether the previous perception was the +45° grating, the −45° grating, or the fusion of two gratings. We did not observe significant difference among different blocks either (Kruskal-Wallis test, p>0.5). Thus, the results of the brief-presentation experiment are not affected by the sensory memory.
